# Cortical interneuron development is affected in 4H leukodystrophy

**DOI:** 10.1093/brain/awad017

**Published:** 2023-02-02

**Authors:** Stephanie Dooves, Liza M L Kok, Dwayne B Holmes, Nicole Breeuwsma, Marjolein Breur, Marianna Bugiani, Nicole I Wolf, Vivi M Heine

**Affiliations:** Department of Complex Trait Genetics, Center for Neurogenomics and Cognitive Research, Vrije Universiteit Amsterdam, Amsterdam Neuroscience, Amsterdam 1081 HV, The Netherlands; Department of Complex Trait Genetics, Center for Neurogenomics and Cognitive Research, Vrije Universiteit Amsterdam, Amsterdam Neuroscience, Amsterdam 1081 HV, The Netherlands; Department of Pediatrics, Emma Children's Hospital, Amsterdam Leukodystrophy Center, Amsterdam University Medical Centers, Vrije Universiteit Amsterdam, Amsterdam Neuroscience, Amsterdam 1081 HV, The Netherlands; Department of Child and Adolescence Psychiatry, Emma Children's Hospital, Amsterdam UMC, Vrije Universiteit Amsterdam, Amsterdam Neuroscience, Amsterdam 1081 HV, The Netherlands; Department of Pathology, Amsterdam University Medical Centers, Vrije Universiteit Amsterdam, Amsterdam Neuroscience, Amsterdam 1081 HV, The Netherlands; Department of Pathology, Amsterdam University Medical Centers, Vrije Universiteit Amsterdam, Amsterdam Neuroscience, Amsterdam 1081 HV, The Netherlands; Department of Pediatrics, Emma Children's Hospital, Amsterdam Leukodystrophy Center, Amsterdam University Medical Centers, Vrije Universiteit Amsterdam, Amsterdam Neuroscience, Amsterdam 1081 HV, The Netherlands; Department of Complex Trait Genetics, Center for Neurogenomics and Cognitive Research, Vrije Universiteit Amsterdam, Amsterdam Neuroscience, Amsterdam 1081 HV, The Netherlands; Department of Child and Adolescence Psychiatry, Emma Children's Hospital, Amsterdam UMC, Vrije Universiteit Amsterdam, Amsterdam Neuroscience, Amsterdam 1081 HV, The Netherlands

**Keywords:** 4H leukodystrophy, POLR3, ARX, iPSC, interneuron, cortex

## Abstract

4H leukodystrophy is a rare genetic disorder classically characterized by hypomyelination, hypodontia and hypogonadotropic hypogonadism. With the discovery that 4H is caused by mutations that affect RNA polymerase III, mainly involved in the transcription of small non-coding RNAs, patients with atypical presentations with mainly a neuronal phenotype were also identified. Pathomechanisms of 4H brain abnormalities are still unknown and research is hampered by a lack of preclinical models. We aimed to identify cells and pathways that are affected by 4H mutations using induced pluripotent stem cell models.

RNA sequencing analysis on induced pluripotent stem cell-derived cerebellar cells revealed several differentially expressed genes between 4H patients and control samples, including reduced *ARX* expression. As *ARX* is involved in early brain and interneuron development, we studied and confirmed interneuron changes in primary tissue of 4H patients. Subsequently, we studied interneuron changes in more depth and analysed induced pluripotent stem cell-derived cortical neuron cultures for changes in neuronal morphology, synaptic balance, network activity and myelination. We showed a decreased percentage of GABAergic synapses in 4H, which correlated to increased neuronal network activity. Treatment of cultures with GABA antagonists led to a significant increase in neuronal network activity in control cells but not in 4H cells, also pointing to lack of inhibitory activity in 4H. Myelination and oligodendrocyte maturation in cultures with 4H neurons was normal, and treatment with sonic hedgehog agonist SAG did not improve 4H related neuronal phenotypes. Quantitative PCR analysis revealed increased expression of parvalbumin interneuron marker *ERBB4*, suggesting that the development rather than generation of interneurons may be affected in 4H.

Together, these results indicate that interneurons are involved, possibly parvalbumin interneurons, in disease mechanisms of 4H leukodystrophy.

## Teaser

4H leukodystrophy is a rare white matter disorder with unknown pathomechanisms. Using iPSC-derived cultures, Dooves *et al*. reveal reduced expression of ARX, increased neuronal network activity, and altered development of interneurons—particularly parvalbumin lineage—in patients with this disorder.

## Introduction

Leukodystrophies are genetic disorders characterized by primary brain white matter involvement. In children, leukodystrophies are often progressive and can lead to early death.^1^ Until several years ago, up to half of patients with leukodystrophies did not receive a genetic diagnosis. Since, developments in next generation sequencing techniques have led to the rapid identification of gene mutations underlying different childhood leukodystrophies, such as *de novo* mutations in structural genes, e.g. *TUBB4A*, in hypomyelination with atrophy of the basal ganglia and cerebellum (OMIM 612438) or mutations in genes encoding proteins essential for translation, e.g. *DARS* (OMIM 615281) or *RARS* (OMIM 616140) in hypomyelinating leukodystrophies.^2–5^ Insights into genetic causes allowed incredible progress in diagnostics, but our understanding of the mechanisms responsible for disease pathology is still lacking.^6,7^

One of the more prevalent leukodystrophies is 4H syndrome (OMIM 612440), originally characterized by hypomyelination, hypogonadotropic hypogonadism and hypodontia.^8–10^ Other characteristics are cerebellar atrophy and myopia. Epilepsy has been described in some patients. Whole exome sequencing has revealed that 4H leukodystrophy is caused by abnormal RNA polymerase III (POLR3), and so far variants in genes encoding different POLR3 subunits, *POLR3A*, *POLR3B*, *POLR1C* and *POLR3K*, have been identified.^11–14^ POLR3 is responsible for the transcription of many different classes of non-protein coding (nc) RNAs, with diverse biological functions, such as transfer (t), ribosomal (r), small nuclear (sn), small nucleolar (sno) and micro (mi) RNAs.^15^ Given the variety of 4H presentations and the diverse regulatory functions of POLR3 genes, there are many potential pathways and tissue-types to research. Interestingly, some POLR3 mutations do not lead to classic brain white matter defects associated with 4H but rather show a predominant neuronal phenotype with involvement of the basal ganglia.^10,16,17^ Therefore, while 4H was originally described as a typical hypomyelinating disorder, the findings suggest that POLR3 variants could also primarily affect neuronal populations.

The generation of induced pluripotent stem cells (iPSCs) from patient tissue allows for patient-specific disease models starting from early embryonic stage^18^ and therefore provide a promising tool to study rare diseases like 4H leukodystrophy. To get insight into disease mechanisms and identify genes that are dysregulated by 4H mutations, we generated patient iPSC-derived cerebellar and cortical cell populations, including neuron-oligodendrocyte co-cultures and studied them by transcriptome, cellomics and multi-electrode array (MEA) analysis. In patient iPSC-derived cerebellar neurons, we found a significantly lower expression of *ARX*, which is involved in early brain development and severe infantile-onset encephalopathies.^19^ As ARX is associated with cortical interneuron development, we performed immunohistochemistry for GAD65/67 on patient material and confirmed alterations in inhibitory neurons in 4H. So we decided to study the development of cortical neurons in more depth. Indeed, the number of GABAergic synapses was significantly decreased in 4H neuronal cultures, which correlated to an increased neuronal network activity. To further identify the specific subtypes that are affected in 4H, quantitative PCR (qPCR) analysis for the major cortical interneuron populations was performed. Interestingly, the expression of *ERBB4*, important for the development of parvalbumin (PV) interneurons, was significantly increased in 4H neurons. Together, our results show that cortical interneuron development is affected in 4H patients, possibly due to pathway changes involving ARX that may affect development of PV interneurons.

## Material and methods

### Patient consent

The Institutional Research Board of the VU University Medical Center approved the study, and written consent was obtained from all participants in accordance with the Declaration of Helsinki.

### Induced pluripotent stem cell culture

Fibroblasts from anonymous donors and 4H patients were reprogrammed into iPSC lines as described previously.^20^ Briefly, iPSCs were generated using an overexpression of *OCT4*, *SOX2*, *KLF4* and *C-MYC*. IPSC lines were confirmed for pluripotency by immunocytochemistry, PCR, alkaline phosphatase, embryoid body formation assay, karyotyping and/or a pluritest. Human embryonic stem cell line H01 was obtained from WiCELL and used as a control line for RNA sequencing experiments. See Supplementary Table 1 for an overview of the iPSC lines. Control and 4H iPSCs were maintained on a vitronectin coating in TeSR E8 medium. Medium was refreshed daily, and cells were passaged once a week using Gentle Cell Dissociation reagent (StemCell Technologies) according to the manufacturer’s protocol. Cells were split 1:10–1:50 to a new well for further maintenance.

### Differentiation in neural cells

Differentiation into cerebellar granule cell neurons was done using a previously reported protocol, involving minimal factors in an N2 + B27 neural maintenance medium (NMM), modified for a 2D monolayer culture.^20,21^

Cortical neuron differentiation was performed as previously described^22^ using dual SMAD inhibition and consequent maturation of cells into glutamatergic and GABAergic neurons. For myelinating cultures, human iPSCs were differentiated into glial progenitor cells as previously described.^23^ To start a co-culture, Day 18 cortical neurons were plated on a monolayer of rat astrocytes and matured for ∼2 weeks, after which iPSC-derived glial progenitor cells were added to the culture.

See Supplementary material for more details.

### RNA sequencing

Total RNA was isolated from cells using TRIzol and chloroform-isopropanol extraction. After quality control samples were run on an Illumina HiSeq2500 according to manufacturer’s protocol. Sequencing reads were aligned to Humane Genome hg38. Similarity between fibroblast, iPSC and cerebellar cells was analysed, and the data from the iPSC and cerebellar cells were compared to previously published gene expression profiles of two iPSCs and two granule cells. Differentially expressed genes (DEG) between 4H and the control were determined with DESeq2 package in R. See Supplementary material for more details.

### Quantitative PCR

For qPCR analysis, RNA was isolated from neurons using TRIzol-chloroform isolation. After production of cDNA, qPCR was performed using SYBR green according to the manufacturer’s protocol. For DEG analysis, data were normalized for housekeeping gene *EIF4G2*. For interneuron subtype analysis, data were normalized for housekeeping gene *EIF4G2* and general neuronal marker *NEUN*. See Supplementary Table 2 for an overview of the primers used. From the results, the fold change 2^−ΔΔCT^ values were calculated, and the log fold change was used for statistical analysis. Data are shown as relative expression compared with Day 30 control neurons.

### Immunostaining

Cells were fixed for immunostaining using either 4% PFA or methanol:acetone fixation. Primary antibodies (Supplementary Table 3) were incubated overnight, followed by 2-h incubation with secondary antibody and embedding with Fluoromount G.

Cortical tissue was obtained from a control (female, 7 months, cause of death: hypoxic ischaemic encephalopathy) and two 4H patients (Patient 1: female, 16 years, homozygous for a POLR3A variant with comparable signs as donor for iPSC line P4; Patient 2: male, 0 years, homozygous POLR3A variant with a severe prenatal presentation) with informed consent of the family. The tissue from Patient 1 was obtained at autopsy at the Erasmus Medical Center Rotterdam, The Netherlands. The tissue was formalin-fixed paraffin-embedded and staining was done on 5-μm thick sections. Staining was developed with 3,3′-diaminobenzidine (DAB, 1:50, DAKO) and counterstained with haematoxylin.

See Supplementary material for more details about immunostainings.

### Smoothened agonist treatment

Smoothened agonist (SAG; Cayman Chemical Co.) was dissolved in DMSO to a stock concentration of 100 μM. In a pilot experiment, one line of control and one line of 4H neurons were treated with 3 nM, 50 nM, 100 nM or 250 nM SAG either starting from Day 18 or Day 30 on. Based on the pilot experiment a concentration of 100 nM SAG was chosen, with treatment starting from Day 18 until the endpoint of the experiment. SAG or vehicle (1:1000 DMSO) was added twice a week with the medium refreshment.

### Multi-electrode array

Neuronal activity was measured on 24-well MEA plates (Multi Channel Systems 24W300/30G-288). Cultures were measured weekly for 30 min, after which data were analysed with Multiwell-Analyzer software (Multichannel Systems) to determine spike count, burst count and network burst counts from raw data. See Supplementary material for more details.

### Statistical analysis

All experiments were repeated on at least two to three independent cell batches per patient, and the results of those experiments were averaged to obtain one value per iPSC line. MEA data were collected on one batch of neurons per cell line. All analyses were either done automated or blinded. Neuronal morphology, myelination and the number of GAD65/67^+^, CTIP2^+^, OLIG2^+^ and MBP^+^ cells were analysed using Columbus software (Perkin Elmer). Synaptic analyses and the percentage of myelinated axons were determined in ImageJ (imagej.nih.gov/ij) using the NeuronJ and SynaptoCount plugins (Supplementary material). Statistical analyses were done in IBM SPSS statistics software version 26 (IBM). Data were tested for normality with a Shapiro–Wilk test. Significance was tested with independent samples *t*-tests (for parametric data) or a Mann–Whitney U-test (for non-parametric data). To compare the effect of compounds on MEA data or the effect of SAG treatment paired samples *t*-test or Wilcoxon signed rank tests were used. All statistical tests were two-tailed and tested against alpha-level *P* < 0.05.

### Data availability

The authors confirm that the data supporting the findings of this study are available within the article and its Supplementary material.

## Results

### *In vitro* products present with iPSC and cerebellar cell identity

**Figure 1:**
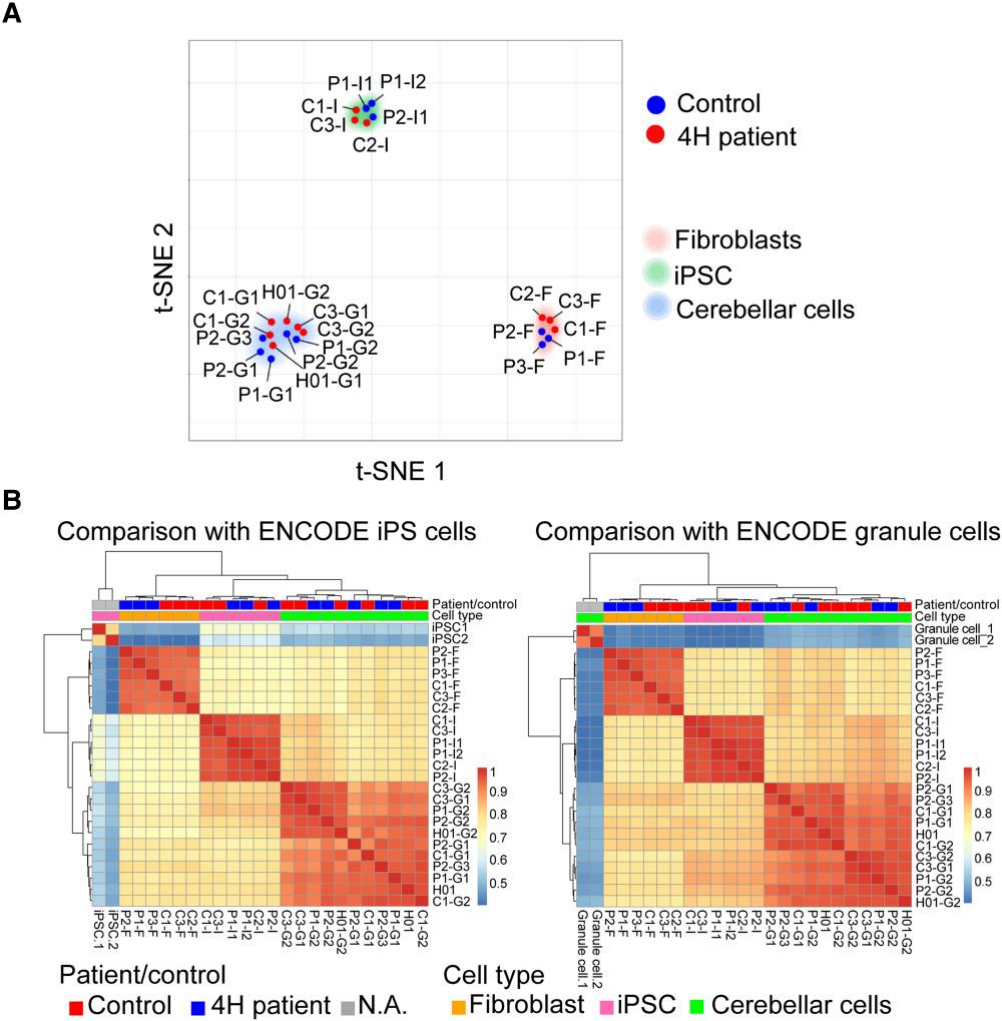
**Reprogramming and differentiation leads to distinct cell types.** (**A**) The projection of 23 samples in 2D space. Each dot represents a sample that is labelled according to patient/control status. Samples are positioned based on relative similarity, showing that fibroblasts, iPSCs and cerebellar cells cluster together. (**B**) Heat maps of the comparison of expression profiles with ENCODE iPSC and granule cell samples. The heat map represents Spearman’s rank correlation coefficient. Patient/control status and cell types are indicated at the *top* of the heat map. C = control; F = fibroblast; G = cerebellar granule cell; I = iPSC; P = patient.

As 4H patients with *POLR3B* variants often have cerebellar atrophy, we differentiated iPSCs into cerebellar neurons as described previously.^21^ We first wanted to establish that reprogramming and differentiation products derived from patient and control fibroblasts were unique cell populations, with relatively little variation due to source material. Further, we tested the validity of the reprogramming protocols by estimating how close each derived cell type resembled published expression profiles. To investigate sample similarity, t-distributed stochastic neighborhood embedding (t-SNE) was performed with perplexity at 5. Cluster analysis showed that samples group together based on cell types (fibroblast, iPSC, product) regardless of cell source (patient or control) (Fig. 1A). The relatively high similarity of samples from the same cell type, regardless of their source, suggests that reprogramming methods were effective at generating a consistent cell type.

To estimate the accuracy of cell reprogramming, pair-wise Spearman’s rank correlations were computed for 23 samples from this study and two expression profiles obtained from ENCODE. One was an ENCODE iPSC expression profile (ENCSR722POQ) and the other an ENCODE granule cell expression profile (ENCSR313IUO), both containing two biological replicates (Fig. 1B). Hierarchical clustering showed that samples in both analyses group by cell type. Colour coding indicates that iPSCs and cerebellar cells are more closely related to the relevant ENCODE profiles than the other cell types (iPSCs = *P* < 0.001; cerebellar cells = *P* < 0.001). Taken together, fibroblast-derived iPSCs and PSC-derived cerebellar cells can be treated as relevant representatives of those developmental stages/lineages for further analyses.

### Transcriptome analysis revealed 4H-associated changes in gene expression

**Figure 2:**
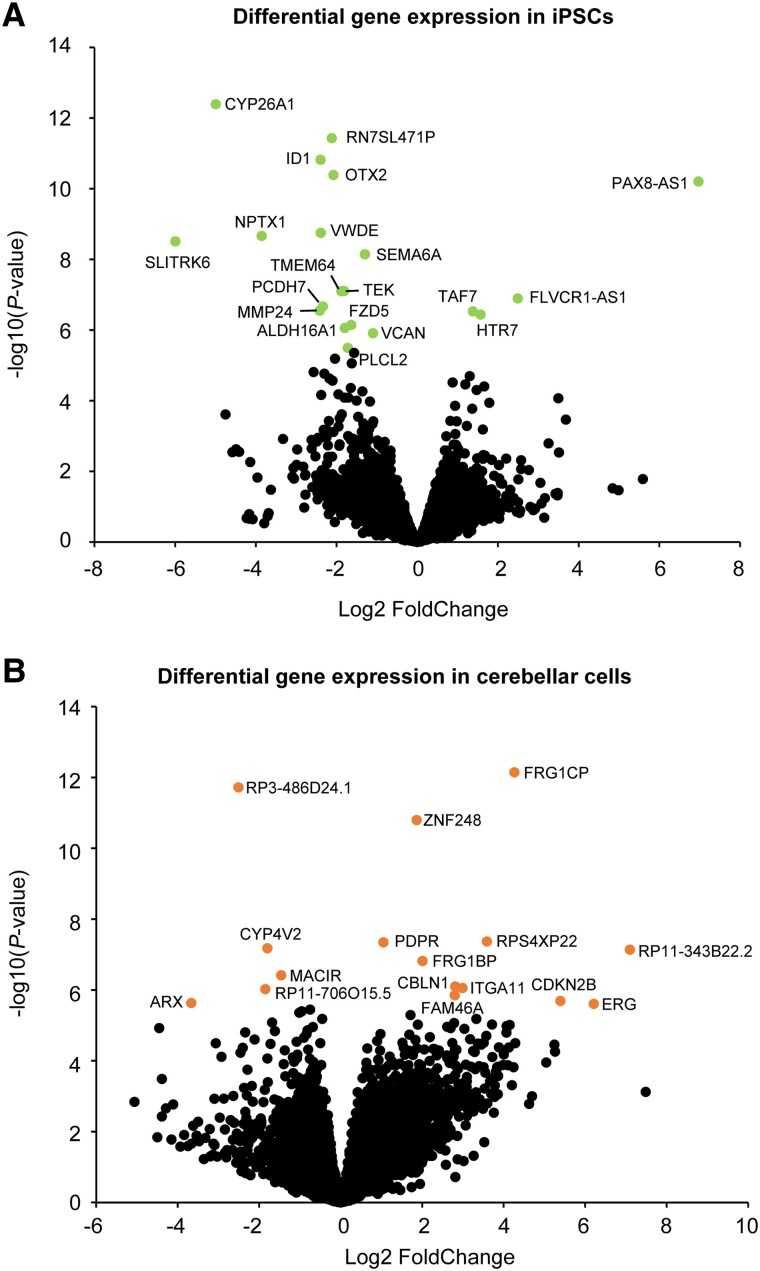
**Differential gene expression reveals genes involved in neurodevelopment.** (**A**) Volcano plot of the RNA sequencing data on patient and control iPSCs. DEGs are visualized by a lighter (green) dot and gene label. (**B**) Volcano plot showing DEGs in 4H cerebellar cells. The significant DEGs are indicated with a lighter (orange) dot and gene label.

RNA sequencing analysis showed several DEGs between 4H patients and controls. In fibroblasts, 29 genes showed a significantly altered expression (Supplementary Table 4), while in iPSCs 20 genes were differentially regulated (Fig. 2A and Supplementary Table 5). Notable DEGs that were downregulated in 4H iPSCs include several genes involved in neural development (*OTX2*, *NPTX1*, *SLITRK6*, *SEMA6A*) and a downregulation of *TMEM64* that plays a role in osteoclast differentiation.^24–28^

In 4H cerebellar cells, 16 genes showed a significant differential expression (Fig. 2B and Supplementary Table 6). 4H cerebellar cells showed an increased expression of *PDPR*, which is associated with intellectual disability^29^ and an increased expression of *CBLN1*, a cerebellum-specific precursor protein.^30^ The downregulation of *ARX* appears to be a potentially significant discovery. *ARX* mutations are associated with several brain disorders, and *ARX* loss of function is associated with abnormal cortical interneuron development and migration.^31–34^

### Cortical interneuron changes in 4H

**Figure 3:**
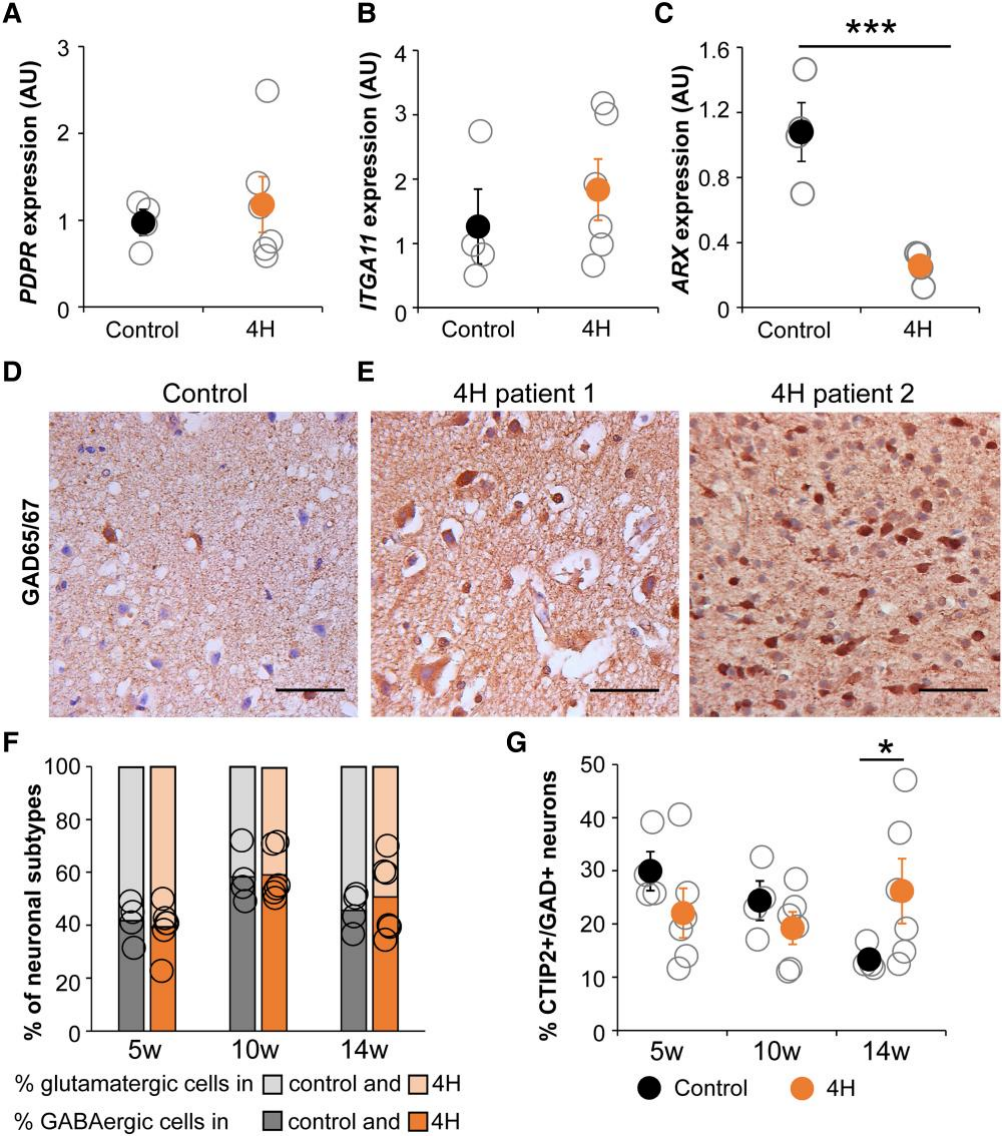
**Cortical interneuron changes in 4H.** (**A**–**C**) Quantitative PCR analysis shows *PDPR*, *ITGA11* and *ARX* expression in 4H and control neurons. RNA was isolated from cortical neurons at Day 18 of the differentiation protocol, which represents an immature state. In 4H neurons, *ARX* expression is significantly downregulated (**C**), while there is no change in *PDPR* or *ITGA11* expression (**A** and **B**). Immunohistochemical analysis for GAD65/67 on primary cortical tissue of a control individual and two 4H patients showed that the pan interneuron marker is increased in 4H brain tissue (**D** and **E**). In iPSC-derived cortical neurons, the proportion of glutamatergic and GABAergic cells (based on GAD65/67 and MAP2 staining) was not changed between control and 4H cells (**F**). A significant increase in the number of CTIP2 and GAD65/67 double positive cells was observed at 14 weeks post-plating (**G**). ***P < 0.001, *P < 0.05, AU = arbitrary units. Open circles represent average per individual patient/control, filled circles represent the mean per genotype. Error bars represent standard error of the mean (SEM). Bars in **F** represent average proportion of GABAergic (dark colours) and glutamatergic (light colours) neurons, with circles representing values of individual lines. Control is shown on the *left*, while data from 4H cultures is shown on the *right* in each panel. Scale bar = 100 µm.

We studied whether the decreased *ARX* expression point to cortical interneuron involvement in 4H. IPSCs were differentiated into cortical neurons, i.e. a mixture of GABAergic interneurons and glutamatergic cortical projection neurons, according to previously established protocols.^22^ At this stage, we were able to include three additional iPSC lines of *POLR3A* patients (P4–P6; Supplementary Table 1). At a neuronal precursor cell state, cells were harvested for RNA analysis. A selected number of DEGs from the cerebellar cells were tested on immature (Day 18) neurons of 4H and controls. No differences in expression levels of *PDPR*, *ITGA11* or *MACIR* (not shown) were observed (Fig. 3A and B). Consistent with the cerebellar cells, *ARX* expression was decreased in neurons of 4H patients [control 1.08 ± 0.31, 4H 0.26 ± 0.10, *t*(6) = 12.073, *P* < 0.001; Fig. 3C], confirming that *ARX* dysregulation may be a common feature in 4H.

To confirm that cortical interneurons are affected in primary brain tissue of 4H patients, we studied patient tissue for expression of GAD65/67, a pan GABAergic marker widely used to visualize all inhibitory neurons. Patient tissue showed elevated immunoreactivity for GAD65/67 compared to control, strengthening the hypothesis that cortical interneuron populations are affected in 4H (control 6% of 1315 cells; 4H Patient 1 42% of 787 cells; 4H Patient 2 36% of 1656 cells; Fig. 3D and E). As patient tissue is extremely scarce and iPSC-based model systems present differential gene expression in 4H cells, we continued functional analysis in iPSC-derived cortical cultures.

The amounts of GABAergic and glutamatergic cells were analysed with staining for GAD65/67 at 5, 10 and 14 weeks post-plating of Day 18 cortical neurons. No changes in the percentages of GABAergic or glutamatergic cells were observed in 4H cultures (Fig. 3F). To test whether there are differences in neuronal maturity, the neurons were stained for CTIP2, which is expressed in interneurons and projection neurons during foetal development.^35–37^ There was an increase in CTIP2^+^ cells in 4H neurons at 14 weeks post-plating, which was significant for the interneuron portion (i.e. CTIP2^+^GAD65/67^+^ cells; control 13.3 ± 2.32, 4H 26.2 ± 13.59, *Z* = −2.13, *P* = 0.033; Fig. 3G) but not for the glutamatergic portion (i.e. CTIP2^+^GAD65/67^−^ cells; control 6.2 ± 2.71, 4H 9.35 ± 4.19). The decrease in *ARX* expression, increased GAD65/67immunoreactivity in 4H patient tissue and sustained CTIP2 positivity in iPSC-derived GABAergic cells together suggests altered interneuron development in 4H.

### 4H neurons show a decreased generation of GABAergic synapses

**Figure 4:**
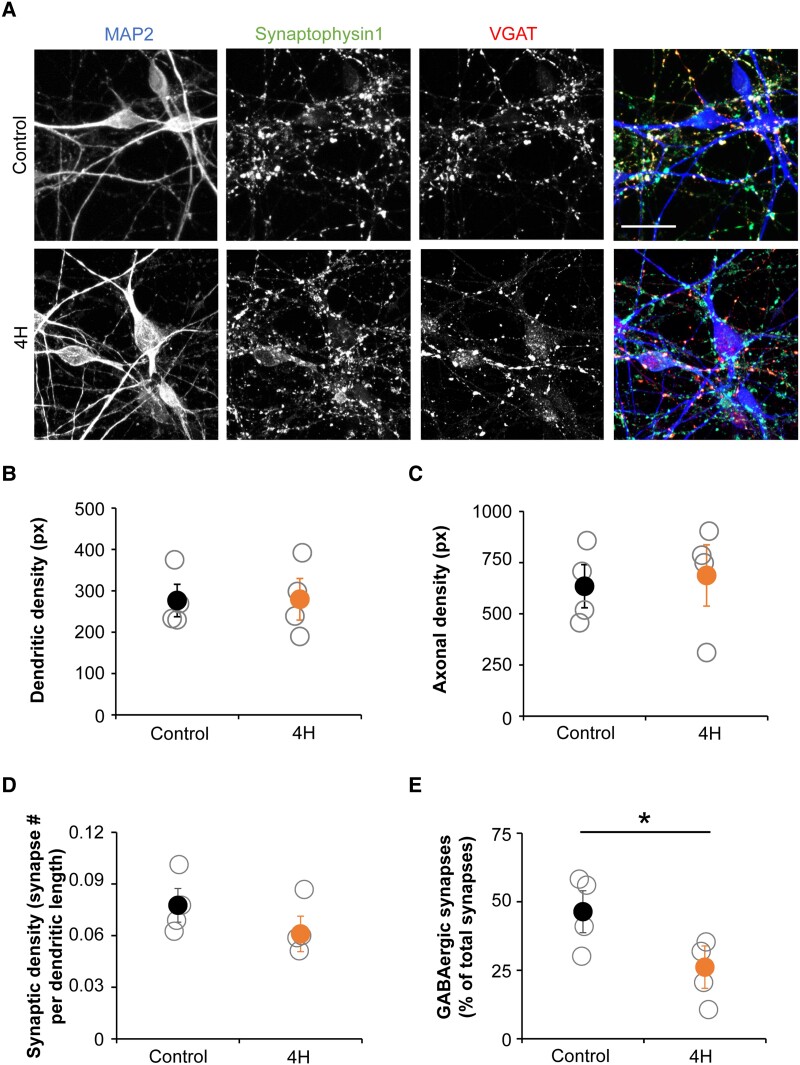
**Cortical neurons of 4H patients show a decreased generation of GABAergic synapses.** (**A**) Representative images of immunostainings on control and 4H neurons. Neurons are stained for dendrites (MAP2, *left*, blue), all synapses (Synaptophysin1, *middle left*, green) and GABAergic synapses (VGAT, *middle right*, red). Dendritic (**B**) and axonal (**C**) densities were not changed in 4H neurons compared to controls. The number of synapses per dendritic length was normal in 4H (**D**), while the percentage of GABAergic synapses was significantly decreased in 4H neurons (**E**). Scale bar = 25 μm, px = pixel, *P < 0.05. (**B**–**E**) Open circles represent average per individual patient/control, filled circles represent the mean per genotype. Error bars represent SEM.

To investigate how cortical neuron development is affected in 4H, cortical neuronal cultures were matured and analysed for morphology changes (Fig. 4). Morphology of 4H and control neurons was analysed on dendritic (MAP2) and axonal (NF-200) staining with automated morphology analysis using Columbus software (Perkin Elmer). The measured parameters included axonal and dendritic length, branching level, and number of extremities and roots. No significant change in any parameter for neuronal morphology was observed between 4H and control neurons (Fig. 4A–C).

To investigate whether 4H cultures present changes in the synapses, we analysed synaptic marker expression by immunocytochemistry (Fig. 4A). In our cortical neuronal cultures, cells differentiate into GABAergic and glutamatergic neurons,^22^ allowing us to study differentiation efficiency into these neuronal subtypes. At Day 56 of differentiation, we studied the total number of synapses by quantifying the number of synaptophysin1 puncta per MAP2 dendritic length. We observed no significant change between the 4H and control cultures (control 0.078 ± 0.017, 4H 0.061 ± 0.018; Fig. 4D). To study whether the fraction of inhibitory synapses changed in 4H cultures, we measured the number of GABAergic presynaptic terminals by co-localization of pre-synaptic protein synaptophysin1 puncta with vesicular GABA transporter (VGAT) puncta. While 4H neurons showed a normal amount of synapses, the percentage of GABAergic synapses was significantly decreased from 46.4% in control to 26.1% in 4H neurons [*t*(6) = 2.503, *P* = 0.046; Fig. 4E]. The decreased generation of GABAergic synapses confirms that interneuron development is affected in 4H.

### 4H neurons show altered network activity

**Figure 5:**
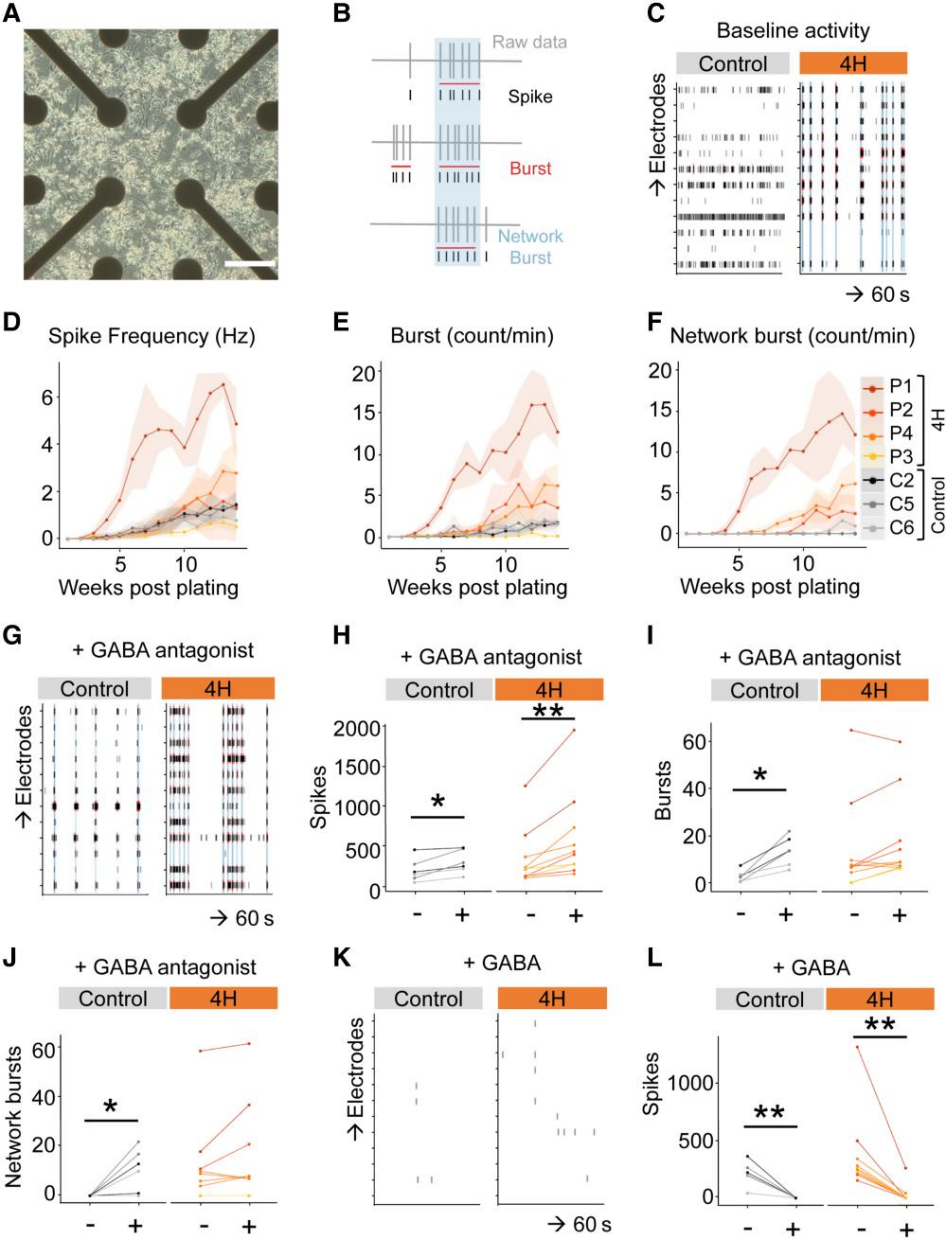
**4H neurons show high network activity and a reduced response to GABA antagonists.** (**A**) Brightfield image of neurons plated on MEA. (**B**) Schematic example of spike and burst detection from MEA recording. (**C**) Representative example of baseline activity at 14 weeks post-plating over 60 s on different electrodes in control and 4H neuronal cultures. Of the 4H neurons three of four show an increased spike frequency (**D**), burst count (**E**) and network burst count (**F**) than control lines, although the differences are not statistically significant. (**G**–**J**) After addition of GABA antagonists bicuculline and gabazine, control neurons show a significantly increase in spikes (**H**), burst (**I**) and network bursts (**J**), while 4H neurons only show an increase in spikes (**I**). (**K** and **L**) Addition of GABA decreased the number of spikes in both control and 4H neurons. Scale bar = 200 μm. *P < 0.05, **P < 0.01. (**D**–**F**) Data-points and lines represent averaged data per patient, with the light areas representing SD per patient. (**H**–**J** and **L**) Data-points and lines represent data per well, with lines with the same colour value representing wells from the same individual.

To assess whether the decreased percentage of GABAergic synapses had consequences for network activity, we recorded spontaneous activity of developing neuronal networks from three controls and four 4H patients plated on MEAs (Fig. 5A). The activity was captured in three parameters namely single spike events, local bursts (spike trains) and network wide bursting events (Fig. 5B). Spontaneous action potential firing in the first weeks mainly consisted of single spike events, while local and network wide bursts occurred as the cultures matured (Fig. 5C–F). Tetrodotoxin administration (TTX, 1 μM) confirmed that baseline activity was dependent on sodium ion channels (Supplementary Fig. 1A and D). At 14 weeks post-plating, three of four 4H patients showed high levels of local and network bursts, which was mostly absent in control cultures (Fig. 5C and F). This is an indication of hyperactivity in 4H networks, although the network activity was not significantly different from control (spikes control 1.19 ± 0.40, 4H 2.41 ± 1.88; bursts control 1.65 ± 0.13, 4H 5.61 ± 5.21; network bursts control 0.33 ± 0.60, 4H 5.17 ± 5.16). Interestingly, the single 4H line (P3) that did not show an increased number of bursts and network bursts was also the 4H line with the highest percentage of GABAergic synapses. A correlation analysis indeed confirmed significant correlations between the percentage of VGAT^+^ synapses and the number of bursts (*r* = −0.774, *P* = 0.041) and network bursts (*r* = −0.786, *P* = 0.036), showing a relation between the amount of GABAergic pre-synapses and network activity.

To establish whether increased network activity in 4H neuronal networks is due to altered synaptic signalling, modulators for glutamate or GABA signalling were added to the cultures at 14 weeks post-plating. Network activity was measured for 10 min before and after addition of modulators. Ionotropic AMPA receptor antagonist DNQX (10 uM) or NMDA antagonist APV (50 µM) were used to measure glutamatergic signalling. The inhibition of either one of these glutamate receptors resulted in a significant decrease of spike count for both 4H and control networks (Supplementary Fig. 1B–C, E and F). In line with the spike counts, burst and network bursts mostly disappeared (data not shown), showing that coordinated network activity depended on glutamatergic signalling and showed no obvious differences between 4H and control lines.

To study GABAergic signalling, GABA_A_ receptor antagonists bicuculline (30 µM) combined with gabazine (20 µM) or the neurotransmitter GABA (10 µM) were administered. After addition, GABA antagonists evoked network bursts in controls that were mostly absent before (burst before 2.85 ± 2.00, after 13.5 ± 5.00, *Z* = −2.201, *P* = 0.028; network burst before 0 ± 0, after 10.5 ± 6.99, *Z* = −2.023, *P* = 0.043; Fig. 5G–J). In contrast, the number of bursts and network bursts did not significantly change in 4H patients (burst before 14.13 ± 12.57, after 18.06 ± 11.60, *Z* = −1.886, *P* = 0.059; network burst before 11.7 ± 10.91, after 15.00 ± 12.45, *Z* = −1.527, *P* = 0.127; Fig. 5G–J). Administration of GABA decreased neuronal activity in both control and 4H cells [control before 215.13 ± 83.50, after 1.68 ± 0.25, *t*(5) = 5.005, *P* = 0.004; 4H before 374.73 ± 212.51, after 32.18 ± 50.59, *Z* = −2.803, *P* = 0.005; Fig. 5K and L], showing normal postsynaptic GABAergic response.

These data shows that the decreased generation of GABAergic synapses correlates to an increase in network activity, and there is a decreased response of 4H neurons to treatment with GABA antagonists.

### 4H neurons show robust myelination *in vitro*

**Figure 6:**
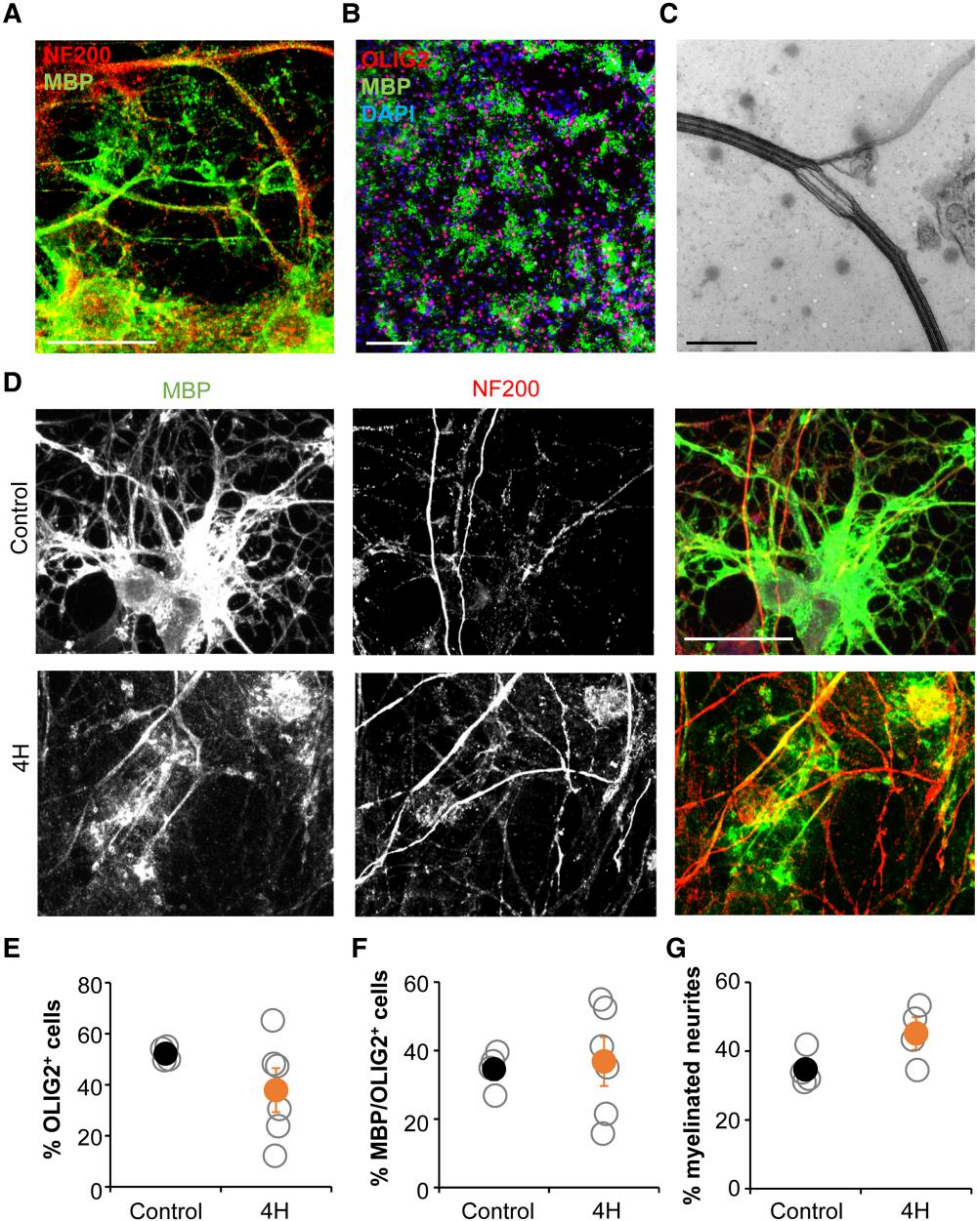
**Oligodendrocyte maturation and myelination in co-culture with 4H neurons is normal. A** and **B** indicate that neuron-oligodendrocyte co-cultures show robust generation of oligodendrocytes and myelination. (**A**) Staining for myelin protein MBP and axonal marker NF200 shows wrapping of oligodendrocyte processes around axons. (**B**) Staining for MBP and early oligodendrocyte marker OLIG2 shows a robust generation and maturation of oligodendrocytes. (**C**) Electron microscopy confirms the presence of compact myelin sheets around neuronal processes. (**D**) Cultures with 4H or control neurons and control oligodendrocytes both show oligodendrocyte maturation and myelination. Quantification showed no changes in oligodendrocyte generation, defined as the percentage of DAPI^+^ cells that were OLIG2^+^ (**E**). Similarly, oligodendrocyte maturation (**F**; percentage of OLIG2^+^ cells that developed in MBP^+^ cells) or the percentage of neurites that is myelinated (**G**) was not changed in cultures with 4H neurons. (**A** and **D**) Scale bar = 25 μm, (**B**) scale bar = 100 μm, (**C**) scale bar = 500 nm. (**E**–**G**) Open circles represent average per individual patient/control, filled circles represent the mean per genotype. Error bars represent SEM.

A subset of human interneurons, mainly the PV neurons, is myelinated^38^ and one of the major characteristics of 4H is hypomyelination. Therefore, we decided to analyse oligodendrocyte maturation and myelination on human neuron-oligodendrocyte co-cultures. We altered a previously published protocol for neuron-OPC culture^23^ to generate a myelinating co-culture. Indeed, after 40 days of co-culture we observed robust generation of mature oligodendrocytes (defined as MBP^+^ cells) and myelination, shown by overlap between MBP and NF200 staining (Fig. 6A and B). The presence of myelin was confirmed by electron microscopy, which showed compacted myelin sheets around neurites (Fig. 6C).

Next, the myelinating cultures were applied to 4H cell lines. Cortical neurons of 4H or control patients were co-cultured with control glial cells. After 40 days of co-culture cells were fixed and stained for neuron (NF200) and oligodendrocyte markers (OLIG2, MBP). In both 4H and control cultures oligodendrocytes matured into MBP^+^ cells and showed myelination of neurites (Fig. 6D). On average there was no statistically significant change in the generation of oligodendrocyte lineage cells in 4H cultures, although three of six patient lines showed a decrease in the percentage of OLIG2^+^ cells compared to controls (control 52.2 ± 2.74; 4H 37.9 ± 19.29; Fig. 6E). In 4H cultures, the percentage of mature oligodendrocytes (MBP^+^/OLIG2^+^ cells) was about 35% for cultures with control and 4H lines, but the variation between 4H lines was high (36.8 ± 15.97), while maturation was quite stable between control lines (34.5 ± 5.50; Fig. 6F). Myelination was analysed on immunostainings by measuring the percentage of NF200^+^ neurites that show co-localization with MBP. No changes in the percentage of myelinated neurites were observed between cultures with 4H and control neurons (control 34.7 ± 4.85; 4H 45.1 ± 8.05; Fig. 6G). While this neuron-glia culture set-up provides a novel tool to study myelination defects in brain diseases, we could not identify changes in 4H.

### Targeting the SHH pathway does not improve 4H interneuron phenotypes

**Figure 7:**
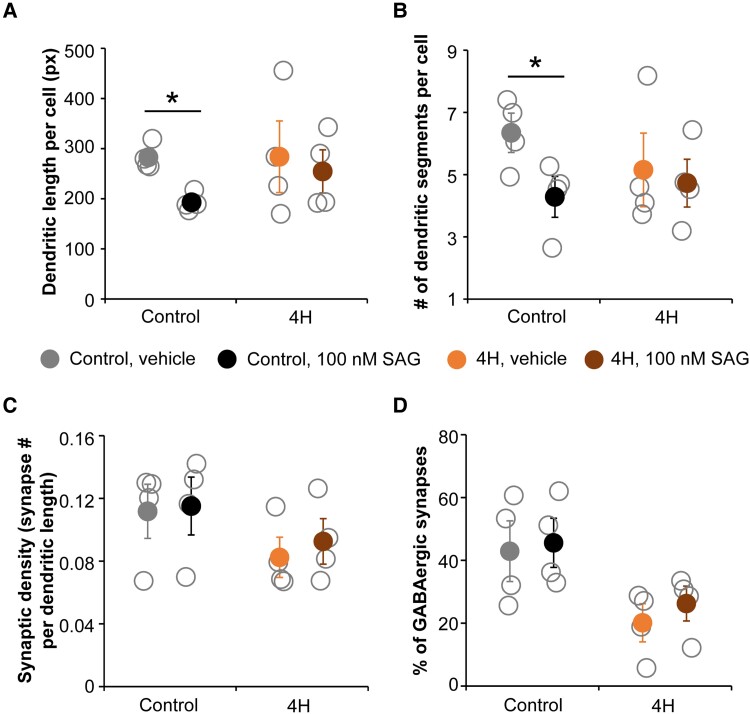
**Targeting the SHH pathway in cortical neurons did not improve 4H associated GABAergic phenotypes.** Control and 4H neurons were treated with vehicle (DMSO) or 100 nM SAG from Day 18 to Day 56. After SAG treatment, the dendritic length per cell (**A**) and number of dendritic segments per cell (**B**), both based on MAP2 staining, were significantly decreased in control but not in 4H neurons. No significant changes in the number of synapses per dendritic length (**C**) or the percentage of GABAergic synapses (**D**) were observed. Px = pixels, *P < 0.05. Open circles represent average per individual patient/control, filled circles represent the mean per genotype. Error bars represent SEM.

ARX is an important regulator of SHH gradients during development,^39^ suggesting that the decreased expression of *ARX* in 4H might affect interneuron development through the SHH pathway. We tested the prospects for targeting the SHH pathway in 4H in the cortical neuronal cultures. In a pilot study it was established that twice a week treatment with SHH pathway agonist SAG at 100 nM from Day 18 onwards increased the percentage of GABAergic synapses on a 4H line, without any (negative) effects on a control line (Supplementary Fig. 2A). PCR analysis confirmed upregulation of SHH target *GLI1* in 4H cells after treatment with 100 nM SAG for 2 weeks (Supplementary Fig. 2B and C). As such, all 4H and iPSC lines were treated with 100 nM SAG from Day 18 until the end point of the experiment at Day 56. Morphology analysis of neurons showed that SAG treatment affected the dendritic complexity by decreasing the number of dendritic segments, extremities and dendritic length per cell in control cells [segments vehicle 6.34 ± 1.07, SAG 4.29 ± 1.12 *t*(6) = 2.481, *P* = 0.024; extremities vehicle 3.34 ± 0.39, SAG 2.36 ± 0.46, *t*(6) = 3.176, *P* = 0.010; length vehicle 283 ± 25, SAG 193 ± 17, *t*(6) = 3.194, *P* = 0.019; Fig. 7A and B]. In 4H cells, no significant effect of SAG treatment on neuronal morphology was observed. A paired samples test comparing vehicle and SAG treatment showed that SAG treatment significantly increased the percentage of VGAT^+^ synapses compared to vehicle treatment [vehicle 31.3 ± 10.19, SAG 34.6 ± 9.93, *t*(20) = −2.125, *P* = 0.046]. However, the increase was small and no significant improvement in the 4H lines specifically was observed (Fig. 7C and D). Although SAG treatment was able to increase expression of SHH target *GLI1*, it was not able to improve 4H interneuron development *in vitro*.

### 4H mutations affect parvalbumin interneuron lineage

**Figure 8:**
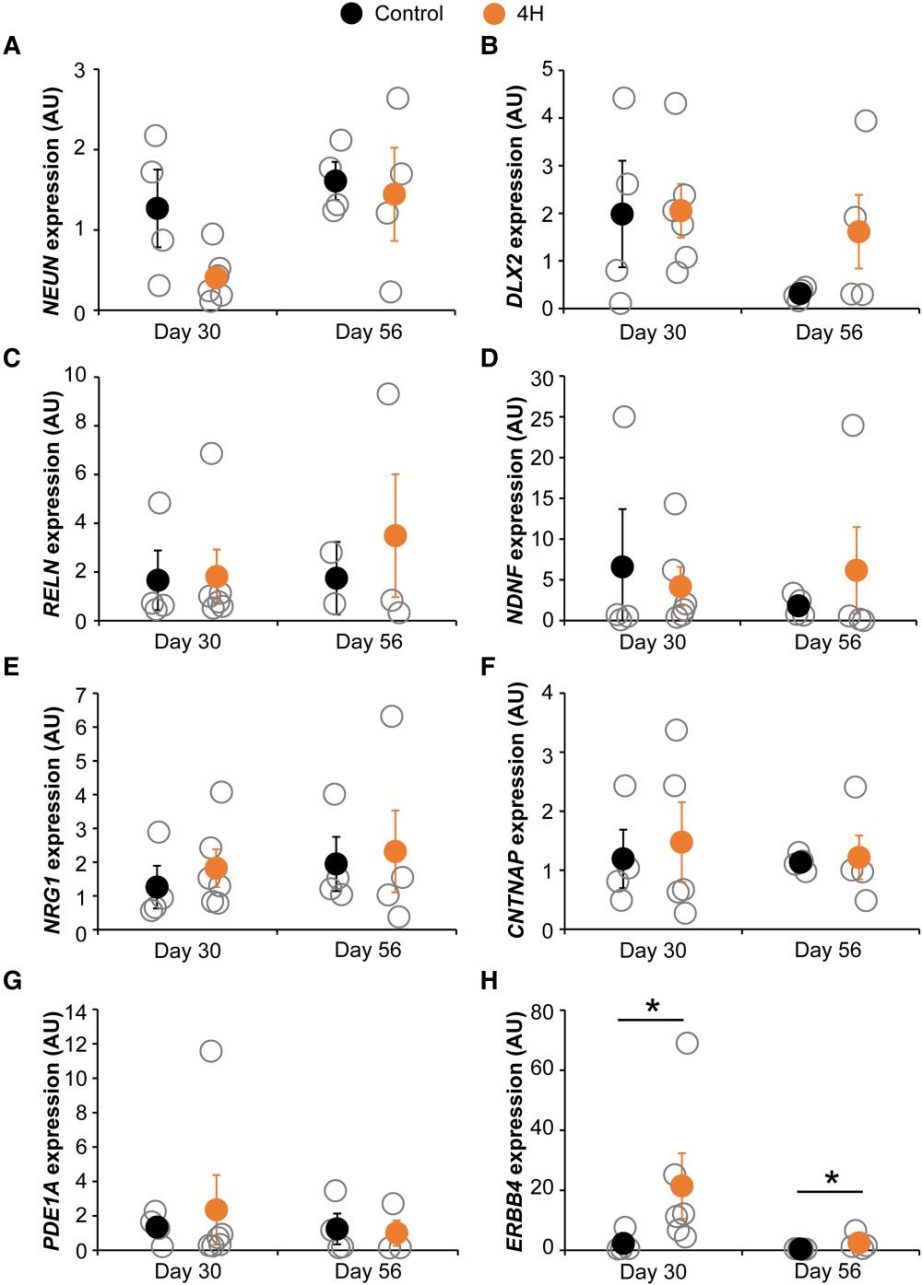
**4H neurons show increased expression of PV interneuron marker *ERBB4*.** QPCR analysis for neuronal and GABAergic markers on Day 30 and Day 56 control and 4H neurons. (**A**) *NEUN* expression, which is decreased in Day 30 4H neurons; therefore, **B**–**H** are corrected for both *NEUN* and *EIF4G2* expression. (**B**) Expression of *DLX2* decreased over time in control neurons, which is less pronounced in 4H cells. (**C**–**H**) QPCR analysis of specific interneuron subtypes: (**C**) *RELN*, an early marker for NDNF interneurons; (**D**) *NDNF*; (**E**) *NRG1*, an early marker for ID2 interneurons; (**F**) *CNTNAP*, an early marker for VIP interneurons; (**G**) *PDE1A*, an early marker for SST interneurons; and (**H**) *ERBB4*, an early marker for PV interneurons. *ERBB4* expression was significantly increased in 4H neurons at Day 30 and Day 56 (**H**). AU = arbitrary units, *P < 0.05.

Next, we aimed to identify whether specific cortical interneuron subtypes are affected in 4H. Primers targeted the five major human interneuron subtypes: somatostatin (SST), PV, VIP, ID2 and NDNF neurons.^40^ The expression of *NEUN* was decreased in 4H cultures at Day 30, although the difference was not significant [control 1.27 ± 0.82, 4H 0.41 ± 0.25, *t*(8) = 2.171, *P* = 0.062; Fig. 8A]. However, since a difference in the number of neurons may affect the relative expression of GABAergic markers when only corrected for a housekeeping gene, all other markers were corrected for expression of both *EIF4G2* and *NEUN*. *DLX2* is important for development of cortical interneurons and downregulated upon maturation of neurons.^41^ Indeed, in control cells the *DLX2* expression decreased between Day 30 and Day 56, which was less pronounced in 4H cells (control Day 30 1.98 ± 1.90, Day 56 0.31 ± 0.12; 4H Day 30 2.06 ± 1.01, Day 56 1.61 ± 1.70; Fig. 8B). Despite the decreased number of GABAergic synapses in 4H cultures, markers for specific interneuron subtypes were not significantly decreased in 4H cultures (Fig. 8C–H). In contrast, the expression of *ERBB4*, a marker for PV neurons, was significantly increased in 4H neurons compared to controls [Day 30 control 2.28 ± 3.47, 4H 21.45 ± 19.51, *Z* = −2.132, *P* = 0.038; Day 56 control 0.42 ± 0.09, 4H 2.50 ± 2.76, *t*(6) = −2.710, *P* = 0.035; Fig. 8H]. This suggests that the generation of specific interneuron subtypes is not impaired in 4H, but rather the development or maturation of interneurons may be affected.

## Discussion

Although the genetic defects causing 4H leukodystrophy have been identified, our knowledge of underlying molecular mechanisms and the affected cellular subtypes is lacking. This study aimed to get more insight into the affected brain cell types and pathways. We started with an unbiased RNA sequencing screen on 4H and control fibroblasts, iPSCs and cerebellar cells. Although only a small number of genes were differentially regulated in all cell types, an interesting finding was the decreased expression of *ARX* in cerebellar cells of 4H patients. Considering the important role of ARX in cortical neuronal development and migration,^32,33,42^ we studied GAD65/67 expression in patient tissue and showed an increase in GAD65/67 immunoreactivity confirming interneuron changes in the cortex of 4H patients. In iPSC-derived cortical neuron cultures, 4H neurons also showed decreased expression of *ARX* and affected interneuron development as measured by a decreased percentage of GABAergic synapses. No changes in the proportion of GABAergic cells were observed, suggesting the change in the number of synapses may be due to affected maturation or synapse formation in 4H neurons. The altered synaptic ratio has functional consequences, as it was correlated to an increased network activity. Decreased GABAergic signalling in 4H also became apparent after treatment with GABA antagonists. 4H neurons did not show changes in activity upon treatment with the GABA antagonists, while control cells showed a significantly increased activity after treatment. As ARX was reported to have important roles in the PV interneurons,^42^ we tested whether specific interneuron lineages were affected in 4H. Indeed qPCR analysis identified an increase in *ERBB4*, a marker for PV neurons, confirming other results that interneuron regulation may be affected in 4H. Together, interneurons are affected in 4H and we show decreased *ARX* expression in different neuronal subtypes using iPSC models. Cortical neuron cultures identified a decreased number of inhibitory synapses, increased network activity, and the results are specifically pointing to defects in PV neurons.

We focused on the involvement of ARX in 4H leukodystrophy because of its role in cortical development and its association with other disorders. Loss of function mutations in ARX lead to pleiotropic disorders such as X-linked lissencephaly with ambigious genitalia (XLAG, OMIM 300215), agenesis of the corpus callosum with abnormal genitalia (OMIM 30004), and lissencephaly with cerebellar hypoplasia.^19^*ARX* impacts interneuron generation, development and migration^43^ and loss of *ARX* expression in interneurons alters their excitability and causes epilepsy in mice.^33,42^ In animal models it was shown that ARX acts with FOXA2 to regulate expression of SHH.^39^ SHH plays important roles in the development of granule cells (and thereby cerebellar volume); oligodendrocytes (and thereby myelination); jaw and teeth; and the eyes.^44–50^ It is possible that POLR3 disorders, and 4H in particular, involve *ARX*-related pathway defects, where altered *ARX* expression drives the clinical phenotype through an ARX to SHH pathway.

Although downregulation of *ARX* may have significant impact on neuronal development, we could not confirm the SHH pathway as a therapeutic target for 4H in our disease model. Neurons were treated with SAG starting from Day 18 to Day 56 of differentiation. At Day 18, the neuronal batches undergo quality control and are frozen for later use. It is possible that treatment starting from Day 18 is too late and some of the developmental alterations following a decreased expression of *ARX* and a dysregulated SHH pathway have already taken place. However, this would mean that targeting the SHH pathway is not a viable option for patients, who are generally diagnosed postnatally. Another explanation may be that the SAG concentration was not high enough to activate the SHH pathway, although the expression of SHH target *GLI1* was increased after SAG treatment. It is possible that the affected development of cortical interneurons is caused by mechanisms that do not involve ARX. In this study we were able to show a correlation between *ARX* levels and interneuron generation, but additional studies are needed to show a causal effect between ARX and 4H interneuron development. Additionally, ARX works on SHH-independent pathways. For example ARX may affect histone demethylation through KDM5C^51,52^ which has been implicated in neurodevelopmental disorders.^53^ In conclusion, SAG treatment did not revert the neuronal phenotype in our cortical 4H cultures and following studies should reveal whether a changed treatment protocol or different target would have more beneficial effects.

The percentage of GABAergic synapses was significantly decreased in 4H cultures, suggesting an alteration in the synaptic balance. Indeed, network activity measurements showed a higher activity in most 4H cultures, although results did not reach statistical significance. Interestingly, there was a significant correlation between the percentage of GABAergic synapses and the amount of bursts and network burst, showing that synaptic balance changes also have consequences for functional network behaviour. This was also confirmed by the measurement of network activity after treatment with GABA antagonists. In control cultures, the network activity significantly increased after blocking GABA-A receptors. However, in 4H cultures treatment with GABA antagonists did not significantly change network activity, suggesting that there was less GABAergic signalling that could respond to antagonistic inhibition. Interestingly, the activity in 4H cultures did decrease after addition of GABA, which suggests a normal postsynaptic GABAergic response in 4H cells. The decreased number of inhibitory synapses could be caused by several mechanisms, e.g. reduced network maturation, a reduction in a specific subset of interneurons, less (active) inhibitory synapses per neuron, or an increase in the number excitatory synapses. Follow-up studies should give more insight into this. Also, as patient lines present differential changes, in line with the broad clinical presentation, an increased panel of patient lines would be advised.

In light of our *in vitro* findings, it seems contradictory that GAD65/67 immunoreactivity is higher in 4H post-mortem tissue. It could possibly be explained if only a subpopulation of interneurons is affected in 4H, as it is reported that interneuron subtypes have different levels of GAD65^54^ or it can be indicative of axonal reorganization of remaining interneurons.^55^ Alternatively, the increased GAD65/67 expression does not reflect changes in the interneuron populations but is rather the consequence of hyperexcitability. Epilepsy is a feature in 4H and human post-mortem studies on temporal lobe epilepsy have identified increased levels of GAD67.^56^ In models of epilepsy, upon neuronal excitotoxicity and increased glutamate release, GAD65/67 is upregulated as compensatory mechanism to convert excess of glutamate into GABA.^57^ If the increase in GAD65/67 immunoreactivity is indeed a secondary effect in 4H this would explain why there is no change in the number of GABAergic cells in the iPSC-derived cultures. However, while post-mortem analysis confirmed network changes in 4H, follow-up studies are warranted.

Alterations in GABAergic activity may underlie 4H associated neurological signs like epilepsy, but also hypomyelination. It is currently unknown how POLR3 mutations cause hypomyelination. Coulombe *et al*.^58^ hypothesized that hypomyelination in 4H is either caused by an unknown POLR3 target that plays a key role in myelin biogenesis, or that POLR3 mutations lead to a globally reduced transcription at a crucial milestone in oligodendrocyte development. We investigated an alternative hypothesis, where the altered myelination is secondary to neuronal dysfunction. Cortical (PV) interneurons in the human brain are myelinated,^38,59^ and myelination can be regulated by GABA receptor activity on oligodendrocytes.^60^ To study whether the decreased interneuron generation in 4H cortical cultures also affected myelination a new co-culture protocol was developed. This co-culture of human neurons and glial cells show robust maturation of oligodendrocytes and myelination of human axons, a challenging phenomenon to study *in vitro*. No differences in oligodendrocyte maturation or myelination were observed between cultures with control or 4H neurons. This could suggest that the hypomyelination observed in 4H patients is caused by an oligodendrocyte intrinsic effect, rather than mediated through affected neurons. However, it is also possible that our culture set-up was not sensitive enough to pick up changes in myelination for 4H. The neurons used for the co-culture consist of a mix of GABAergic and glutamatergic neurons, so a myelination defect on interneurons may be masked by normal myelination of glutamatergic neurons. Additionally, PV neurons mature during late development and not all neurons in the presented co-culture system showed myelinated processes. Although the novel neuron-glia co-culture can measure myelination *in vitro*, further improvement of these model systems may be necessary to identify mechanisms underlying hypomyelination in 4H leukodystrophy.

We aimed to identify the interneuron subtype that was most affected in 4H cultures. We were not able to identify a significant decrease in any of the five major cortical interneurons populations.^40^ Instead, we found a significantly increased expression of *ERBB4*, an early marker for PV interneurons. There are a few possible explanations for the discrepancy between the decrease in GABAergic synapses and the increase in *ERBB4* expression. It is possible that interneuron generation in 4H is normal, but that interneurons are affected during later development or maturation stages. This is consistent with the finding that there were no changes in the number of GAD65/67^+^ cells in 4H cultures. As *ERBB4* is an early marker for PV interneurons, it is also possible that the increase reflects a lack of maturation, rather than an actual increase in PV neurons. A defect in maturation would be consistent with the sustained *DLX2* expression, which decreased in control cells between Day 30 and Day 56, but not in 4H cells. Unfortunately, PV interneurons mature late in cortical development (late gestation/postnatal^61–63^), and we were not able to measure *PVALB* expression in our neuronal cultures. PV interneurons are the largest group of GABAergic neurons in the cortex. They are fast spiking interneurons with a high metabolic demand, which makes them vulnerable for injury.^64^ Interestingly, the development of SST and PV interneurons is depending on SHH levels,^65,66^ and deletion of *Arx* in PV neurons led to increased neuronal activity and altered synaptic properties in a mouse model.^42^ PV interneurons play a crucial role in spike timing of glutamatergic neurons, and abnormalities in PV signalling have been implicated in epilepsy, occasionally seen in 4H patients. For example, optogenic stimulation of PV neurons can induce ictal events in mice^67^ probably due to a depolarization block.^68^ Overall, our data suggest that PV interneurons may be affected specifically in 4H and should be further studied.

To conclude, transcriptome analysis of cerebellar cells differentiated from patient iPSCs revealed ARX as a potentially important regulator of 4H brain pathomechanisms. Indeed, a decreased expression of *ARX* was confirmed in cortical neuron cultures, which showed affected generation of GABAergic synapses consistent with known effects of ARX on interneuron development. The decrease in GABAergic synapses correlated to increased neuronal network activity. QPCR analysis revealed alterations in *ERBB4* expression, suggesting the PV interneurons might be of particular interest for 4H. Although more research is needed, this study provides a first insight into specific brain cell types and pathomechanisms affected in 4H leukodystrophy.

## Supplementary Material

awad017_Supplementary_Data

## References

[1] van der Knaap MS , Schiffmann R, Mochel F, Wolf NI. Diagnosis, prognosis, and treatment of leukodystrophies. Lancet Neurol. 2019;18:962–972. 31307818 10.1016/S1474-4422(19)30143-7

[2] Simons C , Wolf NI, McNeil N, et al A de novo mutation in the beta-tubulin gene TUBB4A results in the leukoencephalopathy hypomyelination with atrophy of the basal ganglia and cerebellum. Am J Hum Genet. 2013;92:767–773. 23582646 10.1016/j.ajhg.2013.03.018 PMC3644625

[3] Wolf NI , Ffrench-Constant C, van der Knaap MS. Hypomyelinating leukodystrophies—Unravelling myelin biology. Nat Rev Neurol. 2021;17:88–103. 33324001 10.1038/s41582-020-00432-1

[4] Taft RJ , Vanderver A, Leventer RJ, et al Mutations in DARS cause hypomyelination with brain stem and spinal cord involvement and leg spasticity. Am J Hum Genet. 2013;92:774–780. 23643384 10.1016/j.ajhg.2013.04.006 PMC3644624

[5] Wolf NI , Salomons GS, Rodenburg RJ, et al Mutations in RARS cause hypomyelination. Ann Neurol. 2014;76:134–139. 24777941 10.1002/ana.24167

[6] Helman G , Van Haren K, Bonkowsky JL, et al Disease specific therapies in leukodystrophies and leukoencephalopathies. Mol Genet Metab. 2015;114:527–536. 25684057 10.1016/j.ymgme.2015.01.014 PMC4390468

[7] van der Knaap MS , Wolf NI, Heine VM. Leukodystrophies: Five new things. Neurol Clin Pract. 2016;6:506–514. 29849248 10.1212/CPJ.0000000000000289 PMC5964825

[8] Schmidt JL , Pizzino A, Nicholl J, et al Estimating the relative frequency of leukodystrophies and recommendations for carrier screening in the era of next-generation sequencing. Am J Med Genet A. 2020;182:1906–1912. 32573057 10.1002/ajmg.a.61641 PMC11348680

[9] Vanderver A , Tonduti D, Bernard G, et al More than hypomyelination in Pol-III disorder. J Neuropathol Exp Neurol. 2013;72:67–75. 23242285 10.1097/NEN.0b013e31827c99d2 PMC3797528

[10] Wolf NI , Vanderver A, van Spaendonk RM, et al Clinical spectrum of 4H leukodystrophy caused by POLR3A and POLR3B mutations. Neurology. 2014;83:1898–1905. 25339210 10.1212/WNL.0000000000001002 PMC4248461

[11] Bernard G , Chouery E, Putorti ML, et al Mutations of POLR3A encoding a catalytic subunit of RNA polymerase Pol III cause a recessive hypomyelinating leukodystrophy. Am J Hum Genet. 2011;89:415–423. 21855841 10.1016/j.ajhg.2011.07.014 PMC3169829

[12] Tetreault M , Choquet K, Orcesi S, et al Recessive mutations in POLR3B, encoding the second largest subunit of Pol III, cause a rare hypomyelinating leukodystrophy. Am J Hum Genet. 2011;89:652–655. 22036172 10.1016/j.ajhg.2011.10.006 PMC3213403

[13] Dorboz I , Dumay-Odelot H, Boussaid K, et al Mutation in POLR3K causes hypomyelinating leukodystrophy and abnormal ribosomal RNA regulation. Neurol Genet. 2018;4:e289. 30584594 10.1212/NXG.0000000000000289 PMC6283457

[14] Thiffault I , Wolf NI, Forget D, et al Recessive mutations in POLR1C cause a leukodystrophy by impairing biogenesis of RNA polymerase III. Nat Commun. 2015;6:7623. 26151409 10.1038/ncomms8623 PMC4506509

[15] Dieci G , Fiorino G, Castelnuovo M, Teichmann M, Pagano A. The expanding RNA polymerase III transcriptome. Trends Genet. 2007;23:614–622. 17977614 10.1016/j.tig.2007.09.001

[16] Harting I , Al-Saady M, Krageloh-Mann I, et al POLR3A Variants with striatal involvement and extrapyramidal movement disorder. Neurogenetics. 2020;21:121–133. 31940116 10.1007/s10048-019-00602-4 PMC7064625

[17] La Piana R , Cayami FK, Tran LT, et al Diffuse hypomyelination is not obligate for POLR3-related disorders. Neurology. 2016;86:1622–1626. 27029625 10.1212/WNL.0000000000002612 PMC4844237

[18] Takahashi K , Tanabe K, Ohnuki M, et al Induction of pluripotent stem cells from adult human fibroblasts by defined factors. Cell. 2007;131:861–872. 18035408 10.1016/j.cell.2007.11.019

[19] Shoubridge C , Fullston T, Gecz J. ARX spectrum disorders: Making inroads into the molecular pathology. Hum Mutat. 2010;31:889–900. 20506206 10.1002/humu.21288

[20] Holmes DB , Heine VM. Simplified 3D protocol capable of generating early cortical neuroepithelium. Biol Open. 2017;6:402–406. 28167491 10.1242/bio.021725 PMC5374398

[21] Holmes DB , Heine VM. Streamlined 3D cerebellar differentiation protocol with optional 2D modification. J Vis Exp. 2017;130:e56888. 10.3791/56888 PMC5755539 29286492

[22] Nadadhur AG , Emperador Melero J, Meijer M, et al Multi-level characterization of balanced inhibitory-excitatory cortical neuron network derived from human pluripotent stem cells. PLoS One. 2017;12:e0178533. 10.1371/journal.pone.0178533 PMC5460818 28586384

[23] Dooves S , Nadadhur AG, Gasparotto L, Heine VM. Co-culture of human stem cell derived neurons and oligodendrocyte progenitor cells. Bio Protoc. 2019;9:e3350. 10.21769/BioProtoc.3350 PMC7854112 33654852

[24] Mitsogiannis MD , Little GE, Mitchell KJ. Semaphorin-Plexin signaling influences early ventral telencephalic development and thalamocortical axon guidance. Neural Dev. 2017;12:6. 28438183 10.1186/s13064-017-0083-4 PMC5402653

[25] Boles NC , Hirsch SE, Le S, et al NPTX1 Regulates neural lineage specification from human pluripotent stem cells. Cell Rep. 2014;6:724–736. 24529709 10.1016/j.celrep.2014.01.026

[26] Kim H , Kim T, Jeong BC, et al Tmem64 modulates calcium signaling during RANKL-mediated osteoclast differentiation. Cell Metab. 2013;17:249–260. 23395171 10.1016/j.cmet.2013.01.002 PMC3569742

[27] Sandberg CJ , Vik-Mo EO, Behnan J, Helseth E, Langmoen IA. Transcriptional profiling of adult neural stem-like cells from the human brain. PLoS One. 2014;9:e114739. 10.1371/journal.pone.0114739 PMC4267785 25514637

[28] Mortensen AH , Schade V, Lamonerie T, Camper SA. Deletion of OTX2 in neural ectoderm delays anterior pituitary development. Hum Mol Genet. 2015;24:939–953. 25315894 10.1093/hmg/ddu506 PMC4834879

[29] Bruno LP , Doddato G, Valentino F, et al New candidates for autism/intellectual disability identified by whole-exome sequencing. Int J Mol Sci. 2021;22:13439. 34948243 10.3390/ijms222413439 PMC8707363

[30] Hirai H , Pang Z, Bao D, et al Cbln1 is essential for synaptic integrity and plasticity in the cerebellum. Nat Neurosci. 2005;8:1534–1541. 16234806 10.1038/nn1576

[31] Gecz J , Cloosterman D, Partington M. ARX: A gene for all seasons. Curr Opin Genet Dev. 2006;16:308–316. 16650978 10.1016/j.gde.2006.04.003

[32] Kitamura K , Yanazawa M, Sugiyama N, et al Mutation of ARX causes abnormal development of forebrain and testes in mice and X-linked lissencephaly with abnormal genitalia in humans. Nat Genet. 2002;32:359–369. 12379852 10.1038/ng1009

[33] Marsh ED , Nasrallah MP, Walsh C, et al Developmental interneuron subtype deficits after targeted loss of Arx. BMC Neurosci. 2016;17:35. 27287386 10.1186/s12868-016-0265-8 PMC4902966

[34] Sherr EH . The ARX story (epilepsy, mental retardation, autism, and cerebral malformations): One gene leads to many phenotypes. Curr Opin Pediatr. 2003;15:567–571. 14631200 10.1097/00008480-200312000-00004

[35] Chen B , Wang SS, Hattox AM, Rayburn H, Nelson SB, McConnell SK. The Fezf2-Ctip2 genetic pathway regulates the fate choice of subcortical projection neurons in the developing cerebral cortex. Proc Natl Acad Sci U S A. 2008;105:11382–11387. 18678899 10.1073/pnas.0804918105 PMC2495013

[36] Saito T , Hanai S, Takashima S, et al Neocortical layer formation of human developing brains and lissencephalies: Consideration of layer-specific marker expression. Cereb Cortex. 2011;21:588–596. 20624841 10.1093/cercor/bhq125

[37] Leyva-Díaz E , López-Bendito G. In and out from the cortex: Development of major forebrain connections. Neuroscience. 2013;254:26–44. 24042037 10.1016/j.neuroscience.2013.08.070

[38] Stedehouder J , Couey JJ, Brizee D, et al Fast-spiking parvalbumin interneurons are frequently myelinated in the cerebral cortex of mice and humans. Cereb Cortex. 2017;27:5001–5013. 28922832 10.1093/cercor/bhx203

[39] Cho G , Lim Y, Cho IT, Simonet JC, Golden JA. Arx together with FoxA2, regulates Shh floor plate expression. Dev Biol. 2014;393:137–148. 24968361 10.1016/j.ydbio.2014.06.012 PMC4681535

[40] Yu Y , Zeng Z, Xie D, et al Interneuron origin and molecular diversity in the human fetal brain. Nat Neurosci. 2021;24:1745–1756. 34737447 10.1038/s41593-021-00940-3

[41] Achim K , Salminen M, Partanen J. Mechanisms regulating GABAergic neuron development. Cell Mol Life Sci. 2014;71:1395–1415. 24196748 10.1007/s00018-013-1501-3 PMC11113277

[42] Joseph DJ , Von Deimling M, Hasegawa Y, et al Postnatal Arx transcriptional activity regulates functional properties of PV interneurons. iScience. 2021;24:101999. 10.1016/j.isci.2020.101999 PMC7807163 33490907

[43] Lee K , Ireland K, Bleeze M, Shoubridge C. ARX Polyalanine expansion mutations lead to migration impediment in the rostral cortex coupled with a developmental deficit of calbindin-positive cortical GABAergic interneurons. Neuroscience. 2017;357:220–231. 28627419 10.1016/j.neuroscience.2017.06.010

[44] Dahmane N , Ruiz I, Altaba A. Sonic hedgehog regulates the growth and patterning of the cerebellum. Development. 1999;126:3089–3100. 10375501 10.1242/dev.126.14.3089

[45] Francis-West P , Ladher R, Barlow A, Graveson A. Signalling interactions during facial development. Mech Dev. 1998;75:3–28. 9739099 10.1016/s0925-4773(98)00082-3

[46] Hardcastle Z , Mo R, Hui CC, Sharpe PT. The Shh signalling pathway in tooth development: Defects in Gli2 and Gli3 mutants. Development. 1998;125:2803–2811. 9655803 10.1242/dev.125.15.2803

[47] Kahn BM , Corman TS, Lovelace K, Hong M, Krauss RS, Epstein DJ. Prenatal ethanol exposure in mice phenocopies Cdon mutation by impeding Shh function in the etiology of optic nerve hypoplasia. Dis Model Mech. 2017;10:29–37. 27935818 10.1242/dmm.026195 PMC5278523

[48] Nery S , Wichterle H, Fishell G. Sonic hedgehog contributes to oligodendrocyte specification in the mammalian forebrain. Development. 2001;128:527–540. 11171336 10.1242/dev.128.4.527

[49] Ortega JA , Radonjic NV, Zecevic N. Sonic hedgehog promotes generation and maintenance of human forebrain Olig2 progenitors. Front Cell Neurosci. 2013;7:254. 24379757 10.3389/fncel.2013.00254 PMC3861791

[50] Prykhozhij SV . In the absence of sonic hedgehog, p53 induces apoptosis and inhibits retinal cell proliferation, cell-cycle exit and differentiation in zebrafish. PLoS One. 2010;5:e13549. 10.1371/journal.pone.0013549 PMC2958845 21042410

[51] Poeta L , Fusco F, Drongitis D, et al A regulatory path associated with X-linked intellectual disability and epilepsy links KDM5C to the polyalanine expansions in ARX. Am J Hum Genet. 2013;92:114–125. 23246292 10.1016/j.ajhg.2012.11.008 PMC3542471

[52] Poeta L , Padula A, Lioi MB, van Bokhoven H, Miano MG. Analysis of a set of KDM5C regulatory genes mutated in neurodevelopmental disorders identifies temporal coexpression brain signatures. Genes (Basel). 2021;12:1088. 34356104 10.3390/genes12071088 PMC8305412

[53] Vallianatos CN , Raines B, Porter RS, et al Mutually suppressive roles of KMT2A and KDM5C in behaviour, neuronal structure, and histone H3K4 methylation. Commun Biol. 2020;3:278. 32483278 10.1038/s42003-020-1001-6 PMC7264178

[54] Kajita Y , Mushiake H. Heterogeneous GAD65 expression in subtypes of GABAergic neurons across layers of the cerebral cortex and hippocampus. Original research. Front Behav Neurosci. 2021;15:750869. 10.3389/fnbeh.2021.750869 PMC8595203 34803625

[55] Peng Z , Zhang N, Wei W, et al A reorganized GABAergic circuit in a model of epilepsy: Evidence from optogenetic labeling and stimulation of somatostatin interneurons. J Neurosci. 2013;33:14392–14405. 24005292 10.1523/JNEUROSCI.2045-13.2013 PMC3761049

[56] Neder L , Valente V, Carlotti CG, et al Glutamate NMDA receptor subunit R1 and GAD mRNA expression in human temporal lobe epilepsy. Cell Mol Neurobiol. 2002;22:689–698. 12585688 10.1023/A:1021852907068 PMC11533764

[57] Esclapez M , Houser CR. Up-regulation of GAD65 and GAD67 in remaining hippocampal GABA neurons in a model of temporal lobe epilepsy. J Comp Neurol. 1999;412:488–505. 10441235

[58] Coulombe B , Derksen A, La Piana R, Brais B, Gauthier MS, Bernard G. POLR3-related Leukodystrophy: How do mutations affecting RNA polymerase III subunits cause hypomyelination? Fac Rev. 2021;10:12. 33659930 10.12703/r/10-12 PMC7894263

[59] Micheva KD , Wolman D, Mensh BD, et al A large fraction of neocortical myelin ensheathes axons of local inhibitory neurons. Elife. 2016;5:e15784. 10.7554/eLife.15784 PMC4972537 27383052

[60] Pudasaini S , Friedrich V, Buhrer C, Endesfelder S, Scheuer T, Schmitz T. Postnatal myelination of the immature rat cingulum is regulated by GABAB receptor activity. Dev Neurobiol. 2022;82:16–28. 34605209 10.1002/dneu.22853

[61] Cao QL , Yan XX, Luo XG, Garey LJ. Prenatal development of parvalbumin immunoreactivity in the human striate cortex. Cereb Cortex. 1996;6:620–630. 8670687 10.1093/cercor/6.4.620

[62] Fung SJ , Webster MJ, Sivagnanasundaram S, Duncan C, Elashoff M, Weickert CS. Expression of interneuron markers in the dorsolateral prefrontal cortex of the developing human and in schizophrenia. Am J Psychiatry. 2010;167:1479–1488. 21041246 10.1176/appi.ajp.2010.09060784

[63] Ulfig N . Calcium-binding proteins in the human developing brain. Adv Anat Embryol Cell Biol. 2002;165:III–IIX, 1-92. 12236093

[64] Ruden JB , Dugan LL, Konradi C. Parvalbumin interneuron vulnerability and brain disorders. Neuropsychopharmacology. 2021;46:279–287. 32722660 10.1038/s41386-020-0778-9 PMC7852528

[65] Flandin P , Zhao Y, Vogt D, et al Lhx6 and Lhx8 coordinately induce neuronal expression of Shh that controls the generation of interneuron progenitors. Neuron. 2011;70:939–950. 21658586 10.1016/j.neuron.2011.04.020 PMC3153409

[66] Tyson JA , Goldberg EM, Maroof AM, Xu Q, Petros TJ, Anderson SA. Duration of culture and sonic hedgehog signaling differentially specify PV versus SST cortical interneuron fates from embryonic stem cells. Development. 2015;142:1267–1278. 25804737 10.1242/dev.111526 PMC4378243

[67] de Curtis M , Avoli M. GABAergic networks jump-start focal seizures. Epilepsia. 2016;57:679–687. 27061793 10.1111/epi.13370 PMC4878883

[68] Calin A , Ilie AS, Akerman CJ. Disrupting epileptiform activity by preventing parvalbumin interneuron depolarization block. J Neurosci. 2021;41:9452–9465. 34611025 10.1523/JNEUROSCI.1002-20.2021 PMC8580142

